# Thriving at Work Among Clinical Nurses: A Systematic Review and Meta‐Analysis

**DOI:** 10.1155/jonm/9263276

**Published:** 2026-10-28

**Authors:** Fei Guo, Wei Zhao, Wen-Nv Hao

**Affiliations:** ^1^ School of Nursing Inner Mongolia Medical University Hohhot 010059 China immu.edu.cn; ^2^ Department of Emergency Affiliated Hospital of Inner Mongolia Medical University No. 1 Tongdao North Road, Huimin District Hohhot 010050 Inner Mongolia China nmgfy.com

**Keywords:** cross-sectional studies, meta-analysis, nurses, systematic review, thriving at work

## Abstract

**Aim:**

This systematic review aimed to synthesize the available evidence on the level of thriving at work among clinical nurses, assess its overall level, and provide an evidence‐based reference for improving nurses’ occupational psychological well‐being and optimizing nursing management practices.

**Background:**

The global nursing shortage and high turnover rates necessitate a deeper understanding of factors that support nurse retention. Thriving at work is a positive psychological state linked to greater occupational well‐being and lower turnover intention, yet a comprehensive synthesis of the level of thriving at work among clinical nurses is lacking.

**Evaluation:**

A systematic literature search was conducted in eight databases, including PubMed, Web of Science, Embase, Cochrane Library, China National Knowledge Infrastructure (CNKI), Wanfang Data, VIP, and Chinese Biomedical Literature Database (CBM), from database inception to September 23, 2026. Two reviewers independently screened records, extracted data, and assessed methodological quality using the quality assessment tool for cross‐sectional studies recommended by the Agency for Healthcare Research and Quality (AHRQ). Meta‐analyses were performed using Stata 17.0. The percent of maximum possible (POMP) scores were used to standardize scores across different Likert scale versions of the instrument, with POMP‐standardized means and 95% confidence intervals (CIs) as the effect size. Subgroup analyses and meta‐regression were conducted to explore sources of heterogeneity, along with sensitivity analyses and publication bias tests.

**Key Issues:**

A total of 64 cross‐sectional studies were included. Meta‐analysis showed that the pooled POMP score for overall thriving at work among clinical nurses was 0.66 (95% CI: 0.64–0.69), with a 95% prediction interval of 0.45–0.87. The pooled scores for the vitality and learning dimensions were 0.65 (95% CI: 0.62–0.67) and 0.69 (95% CI: 0.65–0.72), respectively. High heterogeneity was observed across studies (*I* ^2^ = 99.7%). Subgroup analyses showed some numerical differences in pooled estimates among subgroups, such as lower values for the central region, other specialized departments, and critical care departments, and slightly higher values for studies using a 5‐point Likert scale and those published in 2025–2026. However, none of these differences were statistically significant (all *p* > 0.05). Univariate meta‐regression showed that geographical region was a significant source of heterogeneity (*p* = 0.017). These subgroup and meta‐regression findings should be considered exploratory.

**Conclusion:**

Meta‐analysis showed that the pooled POMP score for thriving at work among clinical nurses was 0.66, corresponding to 66% of the maximum possible score, indicating room for improvement. The learning dimension scored higher than the vitality dimension, suggesting strong professional growth motivation but relatively insufficient work vitality. Exploratory findings from some subgroup analyses may inform future research. Given the extremely high heterogeneity among included studies, the pooled estimate should be interpreted with caution.

**Implications for Nursing Management:**

These findings suggest that nursing managers should prioritize the level of thriving at work among clinical nurses, with particular emphasis on restoring work vitality and maintaining learning motivation. Nursing managers are encouraged to build a supportive organizational work environment across structural, cultural, resource‐related, and relational dimensions to promote nurses’ thriving at work. This may help enhance nurses’ positive occupational psychological state and strengthen nursing workforce stability.

## 1. Introduction

Nurses constitute the largest occupational group in the healthcare workforce and are indispensable in reducing inequities in access to health services and advancing the health‐related Sustainable Development Goals [1]. The International Council of Nurses (ICN) has explicitly stated that strengthening the nursing workforce is not merely a staffing imperative but a prerequisite for quality care and health system resilience globally [2]. However, this pivotal role is accompanied by a professional reality characterized by high‐intensity workloads, deep emotional engagement, and considerable responsibility. As highlighted by previous research, nurses are among the occupational groups at the highest risk of exposure to psychosocial hazards [3]. In their daily work, nurses are not only required to cope with heavy clinical workloads, frequent shift schedules, and various emergencies but are also continuously exposed to multiple psychological stressors, including patient suffering, death, and family anxiety [4]. This prolonged, high‐intensity occupational stress places nurses at widespread risk of burnout and emotional exhaustion [5]. Concurrently, the rising intention to leave among nurses worldwide has intensified the loss of nursing personnel, which has become a critical challenge threatening the sustainable development of healthcare systems globally [6]. Therefore, effectively alleviating nurse burnout and reducing turnover intention has become a critical issue in nursing management.

Within the research paradigm of positive organizational behavior, efforts to improve nurses’ occupational well‐being and strengthen the nursing workforce should not be limited to addressing negative issues such as burnout and stress, but should also focus on activating nurses’ intrinsic positive psychological resources and professional growth motivation. Research has shown that the more positive experiences nurses have at work, the lower their levels of burnout and turnover intention [7]. In this context, the construct of “thriving at work” has garnered considerable attention. Thriving at work was first proposed by Spreitzer et al. [8] in 2005 and refers to a positive psychological state in which individuals simultaneously experience both “vitality” and “learning” in their work. Vitality refers to the sense of being energized and alive that individuals experience in their work, while learning refers to the growth and progress individuals achieve through acquiring new knowledge and skills [9]. Related studies have shown that nurses’ ability to thrive at work is essential for meeting the demands of an evolving healthcare environment and has also been demonstrated to be closely associated with nurses’ development [10]. Porath et al. [11] pointed out that thriving at work can not only positively predict job performance and facilitate employees’ career development but also contribute to improving physical and mental health and reducing burnout. In addition, enhancing nurses’ level of thriving at work can reduce the intention to leave the organization among early‐career nurses, thereby contributing to workforce stability [12, 13]. Therefore, enhancing nurses’ level of thriving at work holds practical significance for improving their well‐being and strengthening workforce stability.

To effectively enhance nurses’ level of thriving at work, it is essential to first accurately assess their current level. In recent years, researchers both domestically and internationally have increasingly focused on thriving at work in the nursing context, and a considerable number of cross‐sectional studies have been conducted among clinical nurses. However, previous studies have reported inconsistent levels of thriving at work among clinical nurses. For example, some studies reported a moderately high level [14], others a moderate level [15], and a study of psychiatric nurses reported a moderately low level [16]. This variability makes it difficult to form a clear and consistent understanding of the overall level of thriving at work among clinical nurses, and the reasons underlying these discrepancies warrant further examination.

Meta‐analysis, as a powerful and objective approach for synthesizing findings from multiple studies, can overcome the limitations of small sample sizes or rare outcomes by pooling results from independent studies, thereby generating more reliable effect estimates [17]. However, to date, no systematic review or meta‐analysis has specifically examined the level of thriving at work among clinical nurses. Therefore, this systematic review and meta‐analysis was conducted to synthesize the existing evidence and comprehensively evaluate the overall level of thriving at work among clinical nurses so as to provide an evidence‐based reference for promoting nurses’ well‐being and stabilizing the nursing workforce.

## 2. Methods

### 2.1. Design

This study strictly adheres to the Preferred Reporting Items for Systematic Reviews and Meta‐Analyses (PRISMA) statement. The research protocol has been registered in the International Prospective Register of Systematic Reviews (PROSPERO) with the registration number CRD420251236104.

### 2.2. Search Strategy

A comprehensive literature search was conducted in the following eight electronic databases: PubMed, Cochrane Library, Web of Science, Embase, China National Knowledge Infrastructure (CNKI), Wanfang Data, VIP Journal Database, and Chinese Biomedical Literature Database (CBM). The search timeframe was from inception to September 23, 2026. A combination of Medical Subject Headings (MeSH) and free‐text terms was employed, combined using Boolean logic operators (AND, OR, NOT), with the search strategy adapted to the specific requirements of each database. Search terms were constructed around “nurses” and “thriving at work” and their related synonyms. The complete search strategies for all databases are provided in Supporting Information Table S1.

### 2.3. Inclusion and Exclusion Criteria

The inclusion criteria were as follows: (1) study type: cross‐sectional studies; (2) study population: clinical nurses; (3) outcome indicators: studies that reported the mean and standard deviation of the total score for thriving at work; (4) language: articles published in Chinese or English; and (5) measurement tool: studies that used the Thriving at Work Scale developed by Porath et al. [11] or its translated and adapted versions.

The exclusion criteria were as follows: (1) duplicate publications; (2) studies from which the required data could not be extracted; (3) studies for which the full text could not be obtained; and (4) reviews, commentaries, conference abstracts, and dissertations.

### 2.4. Study Selection and Data Extraction

Two reviewers independently performed the study selection and data extraction. Before the formal screening process, the two reviewers conducted a pilot screening on five randomly selected articles to align their understanding and application of the inclusion and exclusion criteria. Formal screening commenced after consensus was reached on the pilot screening results. First, duplicate records were removed using NoteExpress software. Next, the reviewers screened the titles and abstracts for initial eligibility, followed by a full‐text review of potentially eligible studies. After the screening was completed, the results were cross‐checked between the two reviewers. Disagreements were resolved through discussion or by consultation with a third reviewer. The following information was extracted from each included study: first author, publication year, sample size, study region, clinical department, study design, scale version, and the mean and standard deviation of the total score for thriving at work and its subdimensions.

### 2.5. Quality Assessment

The quality assessment tool recommended by the Agency for Healthcare Research and Quality (AHRQ) is one of the internationally recognized methodological quality assessment tools for cross‐sectional studies [18]. Unlike the Joanna Briggs Institute (JBI) checklist, which is applicable to multiple study designs, the AHRQ methodological checklist is specifically designed for cross‐sectional studies [19] and has been widely used in systematic reviews of cross‐sectional studies in the nursing field [20]. Given that all included studies in this review employed a cross‐sectional design, the AHRQ criteria were adopted for methodological quality assessment. This tool comprises 11 items, each answered as *yes*, *no*, or *unclear*. A *yes* response is scored as 1 point, while *no* and *unclear* are scored as 0 points. The assessment results were categorized into three quality levels: high quality (8–11 points), moderate quality (4–7 points), and low quality (0–3 points) [21]. Before the formal assessment, the two reviewers conducted a pilot assessment to standardize the interpretation of each AHRQ item, ensuring consistency in the application of the criteria. After completing the formal assessment, the two reviewers calculated the agreement rate item by item using the inter‐rater reliability formula proposed by Miles and Huberman [22], defined as the number of agreements divided by the sum of agreements and disagreements. The 11 AHRQ items served as the unit of analysis to determine the overall percentage agreement between the two reviewers. In cases of disagreement, the two reviewers first sought to resolve discrepancies through discussion and re‐examination of their scoring rationales; if consensus could not be reached, a third reviewer was consulted for final adjudication.

### 2.6. Statistical Analysis

Due to the different versions of the Thriving at Work Scale developed by Porath et al. [11], the Likert response options and scoring ranges varied considerably, and the original means could not be directly compared across studies. Therefore, we used the percent of maximum possible (POMP) approach to transform the original total and subdimension scores into standardized values ranging from 0 to 1 [23]. The POMP score was calculated using the formula POMP = (observed score − minimum possible score)/(maximum possible score − minimum possible score). Means and standard deviations were transformed using the same linear transformation: POMP mean = (original mean − minimum possible score)/(maximum possible score − minimum possible score); POMP standard deviation = original standard deviation/(maximum possible score − minimum possible score). The standard error was obtained by dividing the transformed standard deviation by the square root of the sample size. Each study used the minimum and maximum possible scores corresponding to the specific scale version it employed.

Statistical analyses were performed using Stata 17.0. Pooled effect sizes were expressed as POMP‐standardized mean differences with 95% CIs. Heterogeneity across studies was assessed using the *I* ^2^ statistic. If *p* > 0.1, or *p* < 0.1 with *I* ^2^ ≤ 50%, heterogeneity was considered absent or acceptable, and a fixed‐effects model was used. If *p* < 0.1 with *I* ^2^ > 50%, heterogeneity was considered substantial, and a random‐effects model was used for pooling, with the between‐study variance (*τ* ^2^) estimated using the DerSimonian–Laird (DL) method. If heterogeneity was detected, subgroup analyses and meta‐regression were performed based on sample size, study region, publication year, department, scale version, and career stage to explore potential sources of heterogeneity. To assess the generalizability of the pooled estimates, 95% prediction intervals (PIs) were calculated based on the random‐effects model using the formula proposed by Higgins et al. [24] in order to predict the likely distribution of true effects in future similar studies. Sensitivity analyses were conducted by sequentially omitting each study to examine the robustness of the pooled results. Publication bias was assessed using funnel plots combined with Egger’s linear regression test, with *p* > 0.05 indicating no significant publication bias. If publication bias was present, the trim‐and‐fill method was further applied to evaluate its impact on the pooled estimates.

## 3. Results

### 3.1. Literature Screening Process and Results

A total of 2037 records were initially identified. After duplicates were removed using NoteExpress, 1300 records remained. After screening titles and abstracts, 1141 records were excluded for not meeting the inclusion criteria, leaving 159 records for full‐text retrieval. Of these, 8 full texts could not be obtained, and 151 articles were ultimately assessed in full text. After full‐text review, 87 articles were excluded for not meeting the inclusion criteria, and 64 studies were ultimately included. The literature screening process is shown in Figure 1.

**FIGURE 1:**
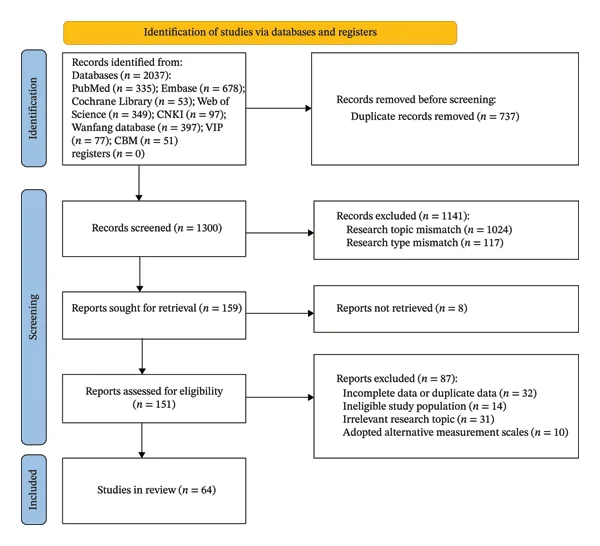
Flowchart of literature screening.

### 3.2. Basic Characteristics of the Included Studies

A total of 64 studies were included, all of which were cross‐sectional in design. The included studies were published between 2016 and 2026, with a total sample size of 33,838 nurses. Of these, 55 studies reported scores for both the vitality and learning dimensions. Geographically, 61 were conducted in China, covering East, Central, and West China, while the remaining 3 were conducted in Belgium, Egypt, and Saudi Arabia, respectively. Regarding measurement instruments, 40 studies used a 10‐item 7‐point Likert scale, 23 used a 10‐item 5‐point Likert scale, and 1 used a 24‐item modified 5‐point Likert scale. In terms of the nurse population, the included studies covered nurses from different clinical departments and career stages. The basic characteristics of the included studies are detailed in Table 1.

**TABLE 1:** Basic characteristics of included studies.

Author, year	Sample size	Study region	Department	Study design	Scale version (Likert format)	Total score of the scale: mean (SD)	Total score of the dimension: mean (SD)	
							Vitality	Learning
He et al., 2023 [25]	1041	Henan, China	Mixed	Cross‐sectional	5‐point Likert (10‐item)	37.96 ± 6.01	18.36 ± 3.23	19.61 ± 3.23
Wang et al., 2025 [26]	441	Jiangsu, China	Mixed	Cross‐sectional	7‐point Likert (10‐item)	47.25 ± 14.16	22.86 ± 7.48	24.38 ± 7.53
Li et al., 2023 [27]	319	Shandong, China	Mixed	Cross‐sectional	7‐point Likert (10‐item)	51.27 ± 8.03	24.89 ± 4.12	26.38 ± 4.42
Yang et al., 2023 [28]	269	Anhui, China	Mixed	Cross‐sectional	7‐point Likert (10‐item)	51.65 ± 10.24	24.68 ± 6.04	26.97 ± 5.07
Wei et al., 2024 [29]	488	Jiangsu, China	Mixed	Cross‐sectional	7‐point Likert (10‐item)	46.42 ± 3.26	23.79 ± 1.93	22.95 ± 2.15
Song et al., 2024 [30]	373	Shandong, China	Mixed	Cross‐sectional	5‐point Likert (10‐item)	36.12 ± 5.78	17.38 ± 3.37	18.74 ± 3.03
Lyu et al., 2025 [31]	291	Beijing and Shandong, China	Mixed	Cross‐sectional	5‐point Likert (10‐item)	35.43 ± 9.05	18.11 ± 4.60	17.32 ± 4.80
Ren et al., 2025 [32]	446	Hubei, China	Mixed	Cross‐sectional	7‐point Likert (10‐item)	50.60 ± 10.11	25.57 ± 6.01	25.03 ± 6.06
Zhu et al., 2022 [15]	427	Shandong, China	Mixed	Cross‐sectional	7‐point Likert (10‐item)	53.67 ± 9.73	25.55 ± 5.83	28.12 ± 4.80
Zhou et al., 2021 [33]	268	Anhui, China	Mixed	Cross‐sectional	7‐point Likert (10‐item)	43.88 ± 9.38	21.84 ± 5.29	22.04 ± 5.22
Fan et al., 2025 [34]	376	Hunan, China	Mixed	Cross‐sectional	7‐point Likert (10‐item)	53.61 ± 9.68	25.49 ± 0.50	28.14 ± 4.79
Jiang et al., 2025 [35]	553	Hunan, China	Mixed	Cross‐sectional	7‐point Likert (10‐item)	42.21 ± 7.36	20.50 ± 4.96	21.71 ± 4.21
Yang et al., 2024 [36]	447	Henan, China	Mixed	Cross‐sectional	7‐point Likert (10‐item)	49.93 ± 10.12	23.91 ± 5.90	26.02 ± 4.87
Zhu et al., 2025 [14]	507	Beijing, China	Mixed	Cross‐sectional	5‐point Likert (10‐item)	39.00 ± 6.98	18.99 ± 4.10	20.01 ± 3.26
Zhang et al., 2023 [37]	114	Xinjiang, China	Reproductive specialist	Cross‐sectional	7‐point Likert (10‐item)	55.44 ± 6.01	26.45 ± 4.64	29.79 ± 4.19
Li et al., 2024 [38]	399	Sichuan, China	Geriatrics	Cross‐sectional	5‐point Likert (10‐item)	39.83 ± 4.71	19.83 ± 2.40	20.01 ± 2.63
Li et al., 2024 [39]	175	Jiangsu, China	Operating room	Cross‐sectional	5‐point Likert (10‐item)	33.28 ± 5.42	15.36 ± 2.27	17.92 ± 2.41
Ma and Chen, 2025 [40]	231	Xinjiang, China	Operating room	Cross‐sectional	7‐point Likert (10‐item)	50.67 ± 10.28	27.82 ± 6.13	22.85 ± 6.21
Ding et al., 2025 [41]	540	Shandong, China	Operating room	Cross‐sectional	7‐point Likert (10‐item)	54.18 ± 7.39	25.06 ± 4.44	29.12 ± 3.93
Li et al., 2025 [42]	289	Shandong, China	Emergency department	Cross‐sectional	7‐point Likert (10‐item)	51.08 ± 8.49	24.72 ± 4.67	26.36 ± 4.65
Liu et al., 2024 [43]	221	Anhui, China	Operating room	Cross‐sectional	7‐point Likert (10‐item)	48.03 ± 7.70	23.32 ± 4.30	24.71 ± 3.98
Li et al., 2023 [44]	312	Shandong, China	Pediatrics	Cross‐sectional	7‐point Likert (10‐item)	37.13 ± 7.52	18.34 ± 3.99	18.79 ± 3.93
Gong et al., 2022 [16]	1629	Hunan, China	Psychiatry	Cross‐sectional	7‐point Likert (10‐item)	36.38 ± 4.77	18.02 ± 2.47	18.36 ± 2.63
Sun et al., 2025 [45]	758	Henan, China	Mixed	Cross‐sectional	5‐point Likert (10‐item)	36.80 ± 8.82	17.64 ± 5.06	19.17 ± 4.17
Lyu et al., 2025 [46]	122	Shandong, China	Pediatric ICU	Cross‐sectional	7‐point Likert (10‐item)	36.22 ± 14.24	NA	NA
Duan et al., 2024 [47]	431	Xinjiang, China	Mixed	Cross‐sectional	5‐point Likert (10‐item)	35.46 ± 6.74	17.35 ± 3.71	18.11 ± 3.48
Wang et al., 2024 [48]	448	Henan, China	Mixed	Cross‐sectional	7‐point Likert (10‐item)	42.27 ± 9.26	20.24 ± 5.16	22.03 ± 4.57
Yang et al., 2025 [49]	1089	Shandong, China	Mixed	Cross‐sectional	5‐point Likert (10‐item)	40.46 ± 6.11	19.52 ± 3.61	20.94 ± 2.94
Yuan et al., 2024 [50]	378	Sichuan, China	Mixed	Cross‐sectional	7‐point Likert (10‐item)	52.60 ± 4.80	26.75 ± 2.69	25.84 ± 3.01
Mortier et al., 2016 [51]	355	Ghent, Belgium	Mixed	Cross‐sectional	5‐point Likert (10‐item)	39.36 ± 4.91	19.26 ± 3.26	20.13 ± 2.57
Atalla et al., 2024 [52]	740	Alexandria, Egypt	ICU	Cross‐sectional	5‐point Likert (24‐item)	86.52 ± 8.47	37.11 ± 3.65	49.41 ± 5.97
Shen and Qiao, 2025 [53]	182	Henan, China	Mixed	Cross‐sectional	5‐point Likert (10‐item)	40.13 ± 8.46	16.32 ± 4.67	23.81 ± 5.13
Zhu et al., 2026 [54]	325	Hubei, China	Mixed	Cross‐sectional	5‐point Likert (10‐item)	34.43 ± 4.09	NA	NA
Xia et al., 2026 [55]	392	Sichuan, China	Mixed	Cross‐sectional	7‐point Likert (10‐item)	50.31 ± 8.20	23.48 ± 4.03	23.39 ± 3.68
Tang et al., 2025 [56]	1419	Jiangsu and Henan, China	Mixed	Cross‐sectional	7‐point Likert (10‐item)	55.31 ± 8.04	26.82 ± 4.63	28.49 ± 4.08
Wang et al., 2025 [57]	132	Hunan, China	Critical care	Cross‐sectional	7‐point Likert (10‐item)	51.02 ± 9.11	24.67 ± 4.90	26.35 ± 4.61
Geng et al., 2026 [58]	411	China	Emergency department	Cross‐sectional	7‐point Likert (10‐item)	45.44 ± 10.45	22.70 ± 6.39	22.72 ± 6.59
Ren et al., 2025 [59]	824	Henan, China	Psychiatry	Cross‐sectional	5‐point Likert (10‐item)	29.44 ± 5.95	NA	NA
Liu et al., 2026 [60]	533	Beijing, China	Dental clinic	Cross‐sectional	7‐point Likert (10‐item)	57.22 ± 9.61	27.38 ± 4.71	29.84 ± 4.71
Gu et al., 2026 [61]	186	Guizhou, China	Mixed	Cross‐sectional	5‐point Likert (10‐item)	32.50 ± 6.80	16.10 ± 3.40	16.30 ± 3.20
Yue and Li, 2025 [62]	735	Sichuan, China	Neurology	Cross‐sectional	7‐point Likert (10‐item)	52.22 ± 10.37	24.79 ± 5.97	27.42 ± 5.18
Li et al., 2026 [63]	489	Xinjiang, China	Operating room	Cross‐sectional	7‐point Likert (10‐item)	54.85 ± 9.18	NA	NA
Li et al., 2026 [64]	340	Shandong, China	Mixed	Cross‐sectional	7‐point Likert (10‐item)	55.86 ± 7.80	26.87 ± 4.41	28.99 ± 4.04
Zhu, 2025 [65]	403	Sichuan, China	Hemodialysis unit	Cross‐sectional	5‐point Likert (10‐item)	36.28 ± 4.76	17.81 ± 2.47	18.48 ± 2.92
Li et al., 2025 [66]	511	Tianjin, China	Mixed	Cross‐sectional	5‐point Likert (10‐item)	35.89 ± 5.79	17.17 ± 3.55	18.71 ± 2.96
Chen et al., 2025 [67]	194	Hunan, China	Mixed	Cross‐sectional	5‐point Likert (10‐item)	28.53 ± 5.42	14.79 ± 2.75	13.74 ± 2.51
Su et al., 2026 [68]	595	Tianjin, China	Mixed	Cross‐sectional	7‐point Likert (10‐item)	47.29 ± 7.52	23.42 ± 4.15	23.88 ± 3.70
Zhuang et al., 2026 [69]	401	Zhejiang, China	ICU	Cross‐sectional	7‐point Likert (10‐item)	52.70 ± 7.64	26.10 ± 4.65	26.60 ± 3.77
Chen et al., 2025 [70]	259	Anhui, China	ICU	Cross‐sectional	7‐point Likert (10‐item)	48.69 ± 6.58	24.21 ± 4.07	24.49 ± 3.45
Li et al., 2025 [71]	442	Shaanxi, China	Orthopedics	Cross‐sectional	5‐point Likert (10‐item)	35.70 ± 5.20	18.02 ± 2.89	17.67 ± 2.75
Dai et al., 2026 [72]	1188	Hunan, China	Mixed	Cross‐sectional	7‐point Likert (10‐item)	45.66 ± 7.46	22.86 ± 4.21	22.81 ± 3.97
Ge et al., 2025 [73]	587	Anhui, China	ICU	Cross‐sectional	7‐point Likert (10‐item)	54.33 ± 10.15	NA	NA
Chen et al., 2026 [74]	498	Anhui, China	Mixed	Cross‐sectional	7‐point Likert (10‐item)	49.87 ± 9.01	24.42 ± 4.84	25.45 ± 4.70
Lyu et al., 2026 [75]	715	Shanxi, China	Mixed	Cross‐sectional	7‐point Likert (10‐item)	47.55 ± 11.97	NA	NA
Han et al., 2026 [76]	516	Zhejiang, China	Operating room	Cross‐sectional	7‐point Likert (10‐item)	58.89 ± 8.13	29.35 ± 4.57	29.54 ± 3.96
Zhang et al., 2026 [77]	1007	Shaanxi, China	Mixed	Cross‐sectional	5‐point Likert (10‐item)	39.83 ± 5.78	NA	NA
Zhu et al., 2026 [78]	190	Fujian, China	Dental department	Cross‐sectional	7‐point Likert (10‐item)	52.42 ± 8.89	25.14 ± 5.41	27.28 ± 4.40
Liu et al., 2025 [79]	534	Ningxia, China	Mixed	Cross‐sectional	7‐point Likert (10‐item)	48.06 ± 7.43	23.47 ± 4.38	24.59 ± 4.02
Wu and Liu, 2026 [80]	414	Chongqing, China	Mixed	Cross‐sectional	7‐point Likert (10‐item)	53.94 ± 8.51	26.74 ± 4.67	27.20 ± 4.42
Zhuang et al., 2025 [81]	590	Guangdong, China	Mixed	Cross‐sectional	5‐point Likert (10‐item)	35.75 ± 6.81	16.99 ± 3.76	18.76 ± 3.69
Li et al., 2025 [82]	1311	Fujian, China	Psychiatry	Cross‐sectional	7‐point Likert (10‐item)	46.59 ± 8.53	NA	NA
Xu et al., 2025 [83]	378	Shaanxi, China	ICU	Cross‐sectional	5‐point Likert (10‐item)	38.00 ± 6.19	NA	NA
Zhao et al., 2026 [84]	2337	Shandong, China	Mixed	Cross‐sectional	5‐point Likert (10‐item)	34.91 ± 7.04	17.08 ± 3.74	17.83 ± 3.68
Elsehrawy et al., 2025 [85]	323	Al‐Kharj, Saudi Arabia	Mixed	Cross‐sectional	5‐point Likert (10‐item)	47.06 ± 6.04	23.82 ± 3.57	23.24 ± 4.18

### 3.3. Quality Assessment Results

The AHRQ quality assessment tool was used to evaluate the methodological quality of the 64 included studies. The results showed that 18 studies were rated as high quality (8–11 points), 46 studies as moderate quality (4–7 points), and no studies as low quality. The overall agreement rate between the two reviewers across the 11 AHRQ items was 83.95%, exceeding the 80% acceptability threshold recommended by Miles and Huberman [22], indicating good inter‐rater reliability. The included studies reported insufficiently on continuity of study subjects (Item 4), control of subjective factors (Item 5), reasons for excluding patients (Item 7), handling of missing data (Item 9), and follow‐up (Item 11), which warrants further standardization. Overall, the methodological quality of the included studies was moderate to high. The quality assessment results are provided in Supporting Information Table S2.

### 3.4. Meta‐Analysis Results

#### 3.4.1. Overall Level of Thriving at Work

A total of 64 studies were included in the meta‐analysis. After POMP standardization, the pooled standardized score for overall thriving at work among clinical nurses was calculated. The heterogeneity test showed *I* ^2^ = 99.7%, indicating extremely high heterogeneity among the included studies. Therefore, a random‐effects model was used for quantitative pooling. The pooled analysis showed that the standardized score for thriving at work among clinical nurses was 0.66 (95% CI: 0.64–0.69). The 95% PI based on the random‐effects model was 0.45–0.87. The results are shown in Figure 2.

**FIGURE 2:**
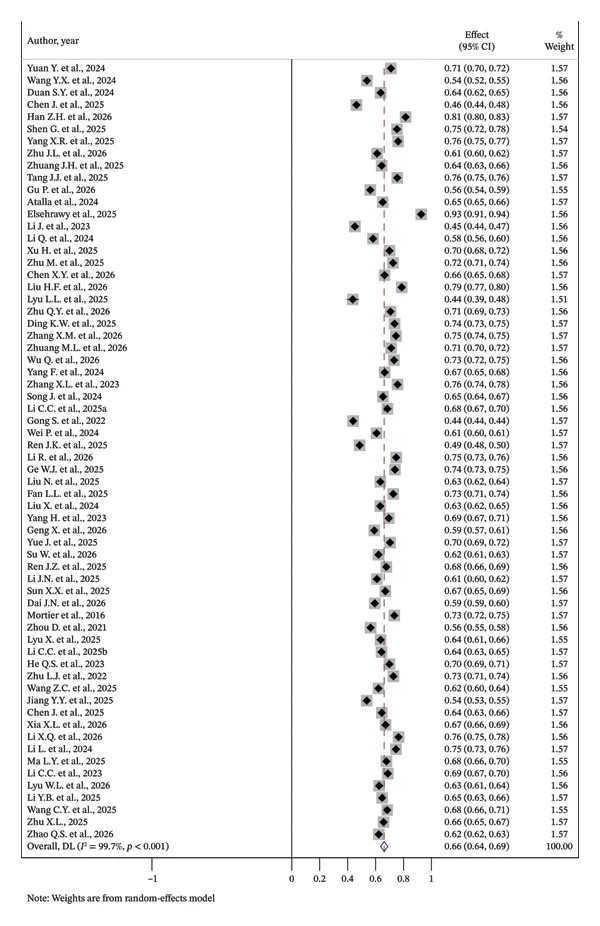
Forest plot of the overall level of thriving at work among clinical nurses.

Among the 64 included studies, 55 reported scores for the learning and vitality dimensions. For the learning dimension, the POMP‐standardized pooled score was 0.69 (95% CI: 0.65–0.72), with a 95% PI of 0.45–0.92. For the vitality dimension, the POMP‐standardized pooled score was 0.65 (95% CI: 0.62–0.67), with a 95% PI of 0.45–0.84. The results are shown in Figures 3 and 4.

**FIGURE 3:**
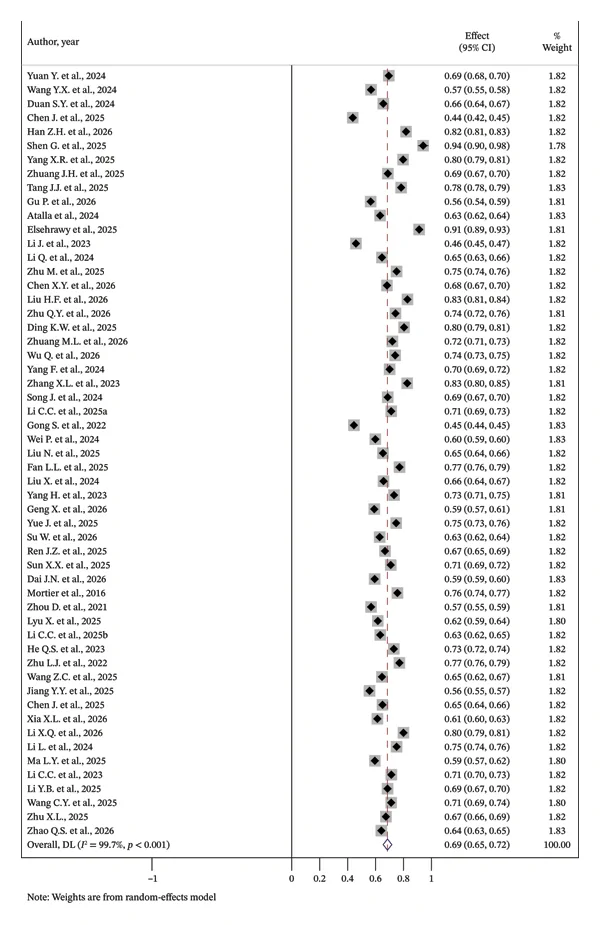
Forest plot of the learning dimension of thriving at work among clinical nurses.

**FIGURE 4:**
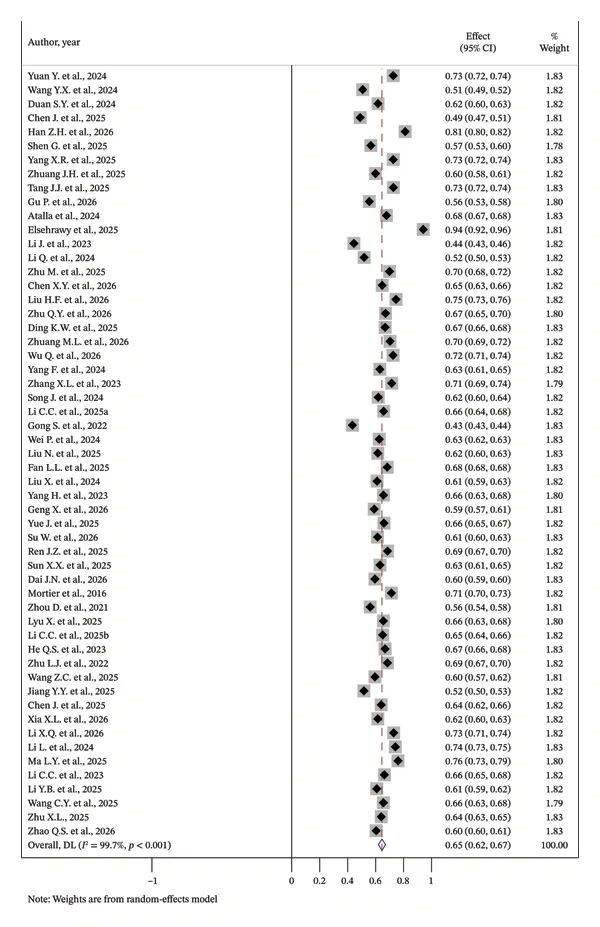
Forest plot of the vitality dimension of thriving at work among clinical nurses.

#### 3.4.2. Subgroup Analysis

Subgroup analyses were performed based on sample size, study region, publication year, department, scale version, and career stage. In the sample size subgroup, the pooled effect size was 0.66 (95% CI: 0.63–0.70, *p* = 0.977) for both the < 400 and ≥ 400 groups. In the study region subgroup, the pooled effect sizes were 0.66 (95% CI: 0.63–0.70) for East China, 0.62 (95% CI: 0.58–0.67) for Central China, 0.69 (95% CI: 0.66–0.71) for West China, and 0.77 (95% CI: 0.63–0.92) for overseas regions (*p* = 0.057). In the publication year subgroup, the pooled effect size was 0.64 (95% CI: 0.59–0.69) for 2016–2024 and 0.67 (95% CI: 0.65–0.69) for 2025–2026 (*p* = 0.330). In the department subgroup, the pooled effect sizes were 0.67 (95% CI: 0.64–0.69) for mixed departments, 0.65 (95% CI: 0.62–0.69) for critical care, 0.70 (95% CI: 0.64–0.76) for operating rooms, and 0.64 (95% CI: 0.55–0.72) for other specialized departments (*p* = 0.546). In the scale version subgroup, the pooled effect sizes were 0.67 (95% CI: 0.63–0.70) for studies using a 5‐point Likert scale and 0.66 (95% CI: 0.62–0.70) for those using a 7‐point Likert scale (*p* = 0.822). For career stage, the pooled effect size was 0.66 for both early‐career nurses (95% CI: 0.59–0.73) and mixed‐seniority nurses (95% CI: 0.63–0.69) (*p* = 0.986). None of these subgroup differences were statistically significant (all *p* > 0.05). The subgroup analysis results are presented in Table 2, and the detailed subgroup forest plots are provided in Supporting Information Figures S1–S6.

**TABLE 2:** Subgroup analysis results.

Subgroup variable	Group	No. of studies	Effect mode	Pooled *r* (95% CI)	Within‐group *I* ^2^ (%)	Weight (%)	*p* between groups
Sample size	< 400	27	Random	0.66 (0.63, 0.70)	99.3	42.06	0.977
	≥ 400	37	Random	0.66 (0.63, 0.70)	99.8	57.94	

Study region	Eastern China	23	Random	0.66 (0.63, 0.70)	99.4	37.07	0.057
	Central China	21	Random	0.62 (0.58, 0.67)	99.7	33.87	
	Western China	15	Random	0.69 (0.66, 0.71)	98.2	24.21	
	Overseas	3	Random	0.77 (0.63, 0.92)	99.8	4.85	

Publication year	2016–2024	19	Random	0.64 (0.59–0.69)	99.8	29.72	0.330
	2025–2026	45	Random	0.67 (0.65–0.69)	99.4	70.28	

Clinical department	Mixed	38	Random	0.67 (0.64, 0.69)	99.4	59.40	0.546
	Critical care	9	Random	0.65 (0.62, 0.69)	98.0	14.01	
	Operating room	6	Random	0.70 (0.64, 0.76)	99.1	9.37	
	Other specialized departments	11	Random	0.64 (0.55, 0.72)	99.9	17.22	

Scale version	5‐point Likert (10‐item)	23	Random	0.67 (0.63–0.70)	99.5	36.51	0.822
	7‐point Likert (10‐item)	40	Random	0.66 (0.62–0.70)	99.8	63.49	

Career stage	Early‐career nurses	8	Random	0.66 (0.59, 0.73)	99.6	12.49	0.986
	Mixed‐seniority nurses	56	Random	0.66 (0.63, 0.69)	99.7	87.51	

Overall		64	Random	0.66 (0.64–0.69)	99.7	100.00	—

#### 3.4.3. Meta‐Regression Analysis

To further explore potential sources of heterogeneity, the six covariates were included in univariate meta‐regression analyses. The results showed that the study region was the only significant moderator (overall *p* = 0.017), explaining 10.81% of the between‐study heterogeneity (*R* ^2^ = 10.81%). Compared with Central China, nurses in overseas regions (*β* = 0.146, *p* = 0.006) and West China (*β* = 0.064, *p* = 0.027) had significantly higher levels of thriving at work, whereas no significant difference was observed between East China and Central China (*β* = 0.039, *p* = 0.132). The remaining covariates (sample size, publication year, department type, scale version, and career stage) did not significantly explain the between‐study heterogeneity (all *p* > 0.05, all *R* ^2^ = 0.00%). The results are shown in Supporting Information Table S3.

### 3.5. Sensitivity Analysis

A leave‐one‐out sensitivity analysis was performed by sequentially omitting each study to assess the robustness of the pooled effect size. The results showed that after omitting any single study, the pooled effect size of the remaining studies ranged from 0.66 to 0.67, and all 95% CIs included the overall pooled estimate (0.66, 95% CI: 0.64–0.69), with no directional change. These findings indicate that the pooled results of this meta‐analysis were robust and were not significantly influenced by any single study (see Supporting Information Figure S7).

### 3.6. Publication Bias

Publication bias was assessed using Egger’s linear regression test. The results showed a bias coefficient of 17.48 (95% CI: 7.66–27.30, *p* = 0.001), suggesting potential publication bias. The funnel plot showed that the distribution of study points was not completely symmetrical (Supporting Information Figure S8). To further evaluate the impact of publication bias on the pooled results, a trim‐and‐fill analysis was performed. The results indicated that 13 studies would need to be imputed, and the adjusted pooled effect size was 0.63 (95% CI: 0.61–0.66), which was consistent in direction with the original result (0.66, 95% CI: 0.64–0.69), suggesting that publication bias had limited substantive impact on the overall conclusions (Supporting Information Figure S9).

## 4. Discussion

### 4.1. Level of Thriving at Work

This meta‐analysis included 64 cross‐sectional studies with a total of 33,838 clinical nurses and comprehensively assessed their level of thriving at work. The pooled POMP‐standardized score was 0.66 (95% CI: 0.64–0.69), corresponding to 66% of the maximum possible score. Given that POMP scores range from 0 to 1, there is still a gap between this score and the maximum possible score (1.0), suggesting room for further improvement. However, given the extremely high heterogeneity across the included studies, the pooled estimate should not be interpreted as a universal benchmark for clinical nurses’ thriving at work but rather as an average derived from highly heterogeneous study populations and settings. The relatively wide PI further suggests that the true level of thriving at work may vary considerably across different clinical contexts. Therefore, when applying these findings to practice or future research, the specific characteristics of the nursing context should be taken into full consideration.

The current level of thriving at work among nurses is likely associated with the combined effects of multiple factors. On the one hand, excessive workloads and unbalanced staffing configurations have placed nurses in a state of both physical and emotional exhaustion, leaving them with little energy to engage in learning and professional growth [86]. On the other hand, against the backdrop of global nurse shortages and increased workloads driven by healthcare reforms, coupled with insufficient social recognition due to the traditional preference for medical over nursing professionals, nurses’ salaries, career development opportunities, and social esteem have failed to match their intense emotional and physical investment. This has created a situation where effort‐reward imbalance and occupational burnout prevail [87], which in turn undermines their vitality and motivation for learning at work. The socially embedded model of thriving at work [8] posits that individual thriving does not arise in isolation but is deeply embedded in the characteristics of the work unit and systematically shaped by organizational contextual factors. The model emphasizes that work unit characteristics and work resources are key factors in promoting employee thriving at work. Therefore, organizational‐level efforts to optimize nurses’ work context and job resources are warranted to promote their thriving at work.

Among the two dimensions of thriving at work, the pooled POMP‐standardized score was 0.69 (95% CI: 0.65–0.72) for the learning dimension and 0.65 (95% CI: 0.62–0.67) for the vitality dimension, with the learning dimension scoring higher than the vitality dimension. This result suggests that nurses generally demonstrate a strong orientation toward knowledge acquisition and skill improvement, reflecting a robust motivation for professional growth among the nursing workforce. However, the consistently lower score in the vitality dimension suggests that nurses lack sufficient work enthusiasm and find it difficult to sustain adequate vigor, which may be associated with energy depletion resulting from long‐term high‐intensity workloads, irregular shift schedules, and highly stressful work environments [88]. Conservation of resources (COR) theory posits that individuals strive to acquire, retain, and protect valued resources, and that resource loss typically exerts a greater impact on individuals than resource gain of equivalent magnitude [89]. When individuals face prolonged high job demands with insufficient supportive resources, resource loss consistently exceeds resource gain, and psychological resources are continuously depleted without effective replenishment, leading to decreased vitality. Learning, by contrast, represents a resource investment behavior. Individuals invest existing resources such as time and energy but expect to obtain future resource gains such as knowledge and skills. Therefore, even when resources are limited, nurses remain willing to invest in learning in exchange for future resource gains. These findings suggest that, while continuing to value professional training, healthcare institutions also need to attend to nurses’ psychological and emotional needs. Adopting a people‐oriented supportive leadership style and fostering a positive organizational culture [90] may help nurses restore their work vitality and sustain their learning motivation.

### 4.2. Subgroup Analyses of Thriving at Work

To explore potential sources of between‐study heterogeneity, subgroup analyses were performed based on sample size, study region, publication year, clinical department, scale version, and career stage, and univariate meta‐regression was further used to examine the explanatory power of each covariate. The meta‐regression results showed that the study region was one of the significant sources of between‐study heterogeneity (*p* = 0.017, *R* ^2^ = 10.81%). However, the overall heterogeneity was still not fully explained, suggesting that it may arise from complex interactions among multiple factors or from unmeasured variables. It should be noted that the statistical power of both univariate meta‐regression and subgroup analyses may be limited by the number of studies [91]. Therefore, these results should be considered exploratory. Although the differences between subgroups were not statistically significant, the numerical differences in effect sizes between subgroups may still provide exploratory reference for future research and warrant further investigation.

The sample‐size subgroup analysis showed identical pooled effect sizes between the < 400 and ≥ 400 groups, with 400 as the cutoff value. Previous methodological studies suggest that sample size is closely related to the stability of pooled estimates in meta‐analyses, and this relationship may not be simply linear; rather, it tends to stabilize after the sample size reaches a certain level, with limited gains in precision from further increases in sample size [92, 93]. Among these, Kent et al. [94], in a methodological study on the stability of contingency table estimates, noted that 400 participants are often regarded as an empirical threshold for reducing sample‐specific variability, and when the sample size reaches or exceeds this level, estimates tend to stabilize. However, this study only used 400 as the cutoff to dichotomize sample size, and whether the results remain stable under different thresholds remains to be verified; moreover, such dichotomization is not equivalent to a continuous examination of the relationship between sample size and effect size. Therefore, these findings should be regarded as hypothesis‐generating only, and future studies could employ alternative grouping strategies or continuous analytical approaches to further examine the association between sample size and the stability of pooled estimates.

The study region emerged as the only significant moderator in meta‐regression (*p* = 0.017, *R* ^2^ = 10.81%). Compared with Central China, overseas regions and West China had significantly higher scores, whereas East China did not differ significantly from Central China. Geographical psychology posits that the geographic region in which individuals reside systematically shapes their thoughts, feelings, and behaviors [95]. The regional numerical variation observed in the present study may be related to differences across regions in healthcare resource allocation, nursing management systems, and sociocultural backgrounds. Previous studies have indicated that nurse staffing is relatively inadequate in Central China and that nurses there experience higher levels of work‐related cumulative fatigue [96]. Given the limited data from West China and overseas regions, as well as the substantial differences in nursing systems across overseas countries, which may affect the precision of subgroup estimates, this finding should be considered exploratory only. Future research could examine, in cross‐cultural comparisons, the influence of macro‐level variables such as regional health system characteristics, organizational context, and cultural values on nurses’ thriving at work.

The results showed that studies published in 2025–2026 had a slightly higher pooled effect size than those published in 2016–2024. Thriving at work is itself a dynamic, context‐dependent psychological state. Spreitzer et al. [8] conceptualized thriving at work as a dynamic psychological state, and this dynamic nature is reflected not only in temporal continuity but also in individuals’ immediate psychological perceptions during ongoing development [97]. Therefore, thriving at work may fluctuate with changes in the healthcare environment and management practices. In recent years, with the promotion of positive organizational behavior in nursing management, increasing attention has been paid to nurses’ positive psychological states. This may have facilitated the implementation of supportive management practices and may be related to the observed numerical differences. Considering that the publication year was divided into only two periods and that all included studies were cross‐sectional, the above findings remain exploratory observations and cannot be used to infer temporal trends or individual‐level dynamic changes. Future longitudinal studies are still needed for further verification.

Our analysis found that operating room nurses reported the highest numerical level of thriving at work, followed by mixed departments, while critical care departments and other specialized departments were relatively lower. The Job characteristics model (JCM) posits that job characteristics such as autonomy, task identity, and feedback influence individuals’ positive work states through experienced meaningfulness, responsibility, and knowledge of results [98]. Emergency department/ICU and other specialized nurses are chronically exposed to high‐pressure and unpredictable work contexts [99], with limited autonomy and task identity. Operating room nurses, by contrast, have more clearly defined, complete, and timely feedback in their work design, and multidisciplinary teamwork may provide some support [100]. These differences in job characteristics may be related to numerical fluctuations in thriving at work, but the above explanations are only exploratory references and should not be overinterpreted. Future research could further examine the potential influence of work design across different departments on nurses’ thriving at work.

The scale version subgroup analysis showed that the pooled effect size reported by studies using a 10‐item 5‐point Likert scale was numerically higher than that reported by studies using a 10‐item 7‐point Likert scale. The number of Likert response options may influence the measurement of thriving at work. Höhne et al. [101] found that the 5‐point scale was more robust to scale direction effects, whereas the 7‐point scale had higher composite reliability. Krosnick et al. [102], based on a comprehensive synthesis of multiple previous studies, noted that 5‐point and 7‐point scales are generally equally optimal, each with its own strengths and weaknesses. Furthermore, translated and cross‐culturally adapted versions may differ in item wording, response dimensions, and sample applicability, and the psychometric properties of the scale may vary accordingly [103]. Therefore, this result is more appropriately considered a psychometric signal, and future research could further examine the measurement equivalence of scales with different numbers of response options and compare their psychometric performance in measuring nurses’ thriving at work.

For career stage, the pooled effect size was identical between early‐career and mixed‐seniority nurses. Chang et al. [104], based on career development stage theory, divided the nursing career into four stages: exploration, establishment, maintenance, and disengagement, and noted that career needs differ across stages. Early‐career nurses are predominantly in the transition from exploration to establishment, while mixed‐seniority nurses are mostly in the establishment or maintenance stage. From the perspective of conservation of resources theory, nurses at different career stages may achieve a similar net effect in resource gain and loss, thereby exhibiting similar levels of thriving at work. However, due to the inconsistent definition of “early‐career” in existing literature and the limited number of studies in this subgroup, potential between‐group differences may have been masked, and the precision of subgroup estimates may have been affected. Therefore, this result should be considered exploratory. Future research could adopt more refined criteria for classifying career stages and focus on the specific support needs during role transitions.

### 4.3. Strengths and Limitations

This study has the following strengths. First, unlike previous meta‐analyses and integrative reviews on thriving at work, this study is the first systematic review and meta‐analysis specifically focusing on the level of thriving at work among clinical nurses. Previous meta‐analyses on thriving at work mainly targeted general working populations in business, education, and management, with a very low proportion of nursing populations. Related reviews in the nursing field mostly focused on the relationship between organizational factors and thriving at work or on specific subgroups and did not quantitatively synthesize the level of thriving at work among clinical nurses. This study fills this evidence gap and provides a quantitative estimate based on a relatively large sample. Second, this study separately pooled the scores of the learning and vitality dimensions, revealing the relative level differences between the two and providing a more precise intervention target for nursing management. Third, it strictly followed the PRISMA statement and was registered in PROSPERO, used POMP standardization to enable cross‐version comparison of scale scores, and conducted subgroup analyses, meta‐regression, sensitivity analyses, and publication bias tests, with relatively rigorous methodology.

Despite these strengths, several limitations should be acknowledged. First, all included studies were cross‐sectional, which precludes establishing causal relationships; the dynamic changes and causal mechanisms of thriving at work still require validation through longitudinal or interventional studies. Second, there was extremely high heterogeneity among the included studies. Although exploratory subgroup analyses and meta‐regression were performed, the heterogeneity could not be fully explained, and caution is needed when interpreting the results of this study. Third, the included studies were concentrated in Asia, predominantly among Chinese nurses, limiting geographical representativeness, and the conclusions should be extrapolated to other regions with caution. Fourth, Egger’s test suggested potential publication bias. Although the trim‐and‐fill method showed a consistent direction of effect size, bias could not be completely ruled out, which may be related to the fact that only publicly available Chinese and English literature was searched and gray literature was not included. Fifth, the number of studies in some subgroups was limited, which may restrict statistical power and robustness; findings based on subgroup comparisons should therefore be considered exploratory.

## 5. Implications for Nursing Management

Thriving at work reflects nurses’ positive psychological state in their professional roles and is closely associated with nursing workforce stability and quality of care. Based on pooled evidence from 64 cross‐sectional studies, this study systematically assessed the overall level of thriving at work among clinical nurses, providing an evidence‐based reference for nursing management practice. Accurate identification of the level and weak links of nurses’ thriving at work can help managers develop targeted strategies to improve nurses’ professional experience.

Implications based on the overall pooled results. Our findings suggest that nurses have strong learning motivation but relatively insufficient work vitality, and the overall level of thriving at work still requires further improvement. Accordingly, nursing managers may focus on three aspects. First, build a supportive organizational work environment. The socially embedded model emphasizes that thriving at work is embedded in the characteristics of the work unit. Therefore, nursing managers can intervene at structural, cultural, resource, and relational levels to enhance nurses’ overall level of thriving at work. At the structural level, enhance nurses’ work autonomy and decision‐making participation; at the cultural level, foster a trusting and inclusive team climate; at the resource level, optimize staffing to reduce workload; and at the relational level, strengthen open communication and support between leaders and nurses. Second, leverage the strengths of the learning dimension. As a knowledge‐intensive and practice‐oriented profession, nursing requires nurses to continuously update knowledge and improve skills. Nurses’ strong motivation for professional growth is an important positive resource for the nursing workforce. Managers should provide clear career development pathways, mentorship, and continuing education opportunities to promote sustained thriving at work among nurses. Third, prioritize the restoration and maintenance of nurses’ work vitality. Lower work vitality may be related to resource depletion caused by long‐term high‐intensity workload, irregular shifts, and high‐pressure environments. Managers should rationally schedule shifts, reduce non‐nursing tasks, and create a recoverable work rhythm for nurses.

Implications based on exploratory subgroup and meta‐regression findings. Exploratory findings from subgroup analyses and meta‐regression may also provide some reference for nursing management. The study region was significant in meta‐regression, explaining a small proportion of between‐study heterogeneity. This suggests that contextual factors such as regional healthcare resource allocation, management systems, and sociocultural backgrounds may be related to nurses’ thriving at work, providing direction for future regionalized research and differentiated support strategies. Although between‐group differences in other subgroups were not statistically significant, their numerical differences still suggest potential variations in thriving at work across study designs, work department characteristics, and career stages, warranting further attention in future research. Exploratory findings at the subgroup level should be interpreted with caution. Future studies could test these findings using more refined subgroup classifications, longitudinal tracking, or interventional designs.

## 6. Conclusion

This systematic review and meta‐analysis comprehensively assessed the level of thriving at work among clinical nurses. The results showed that thriving at work among clinical nurses reached an average of 66% of the maximum possible score, indicating room for improvement. The learning dimension scored higher than the vitality dimension. However, due to the extremely high heterogeneity among studies, this pooled estimate should be interpreted as an overall average across highly heterogeneous study populations and settings and should be interpreted with caution. Meta‐regression indicated that the study region was one of the significant sources of between‐study heterogeneity, and exploratory findings from subgroup analyses may provide reference for future research. These findings suggest that nursing managers may consider providing targeted support at the organizational level. Future research should expand geographical coverage and conduct longitudinal or interventional studies to clarify the dynamic patterns and causal mechanisms of thriving at work, thereby providing evidence‐based support for enhancing nurses’ well‐being and promoting the sustainable development of the nursing workforce.

**Nomenclature**
CNKI: China National Knowledge Infrastructure
CBM: Chinese Biomedical Literature Database
AHRQ: Agency for Healthcare Research and Quality
POMP: Percent of maximum possible
CI: Confidence interval
ICU: Intensive care unit
ICN: International Council of Nurses
PRISMA: Preferred Reporting Items for Systematic Reviews and Meta‐Analyses
PROSPERO: International Prospective Register of Systematic Reviews
MeSH: Medical Subject Headings
JBI: Joanna Briggs Institute
PI: Prediction interval
COR: Conservation of resources
JCM: Job characteristics model

## Author Contributions

Conceptualization: Fei Guo, Wen‐Nv Hao.

Methodology: Fei Guo, Wei Zhao.

Resources: Fei Guo, Wei Zhao.

Investigation: Fei Guo, Wei Zhao.

Data curation: Fei Guo, Wei Zhao.

Formal analysis: Fei Guo.

Supervision: Wen‐Nv Hao.

Project administration: Wen‐Nv Hao.

Writing–original draft preparation: Fei Guo, Wen‐Nv Hao.

Writing–review and editing: Fei Guo, Wen‐Nv Hao.

## Funding

This study was supported by the Graduate Education and Teaching Reform Research Project of the Inner Mongolia Autonomous Region (grant number JG2024058C).

## Conflicts of Interest

The authors declare no conflicts of interest.

## Supporting Information

Additional supporting information can be found online in the Supporting Information section.

## Supporting information


**Supporting Information** The following supporting information is available as part of the online article: Table S1. Search strategies for all databases. Table S2. Quality assessment results of included studies. Table S3. Univariate meta‐regression analysis results. Figure S1. Forest plot of subgroup analysis by sample size. Figure S2. Forest plot of subgroup analysis by study region. Figure S3. Forest plot of subgroup analysis by publication year. Figure S4. Forest plot of subgroup analysis by department. Figure S5. Forest plot of subgroup analysis by scale version. Figure S6. Forest plot of subgroup analysis by career stage. Figure S7. Leave‐one‐out sensitivity analysis. Figure S8. Funnel plot for publication bias assessment. Figure S9. Trim‐and‐fill adjusted funnel plot. PRISMA_2020_checklist.

## Data Availability

The conclusions presented in this review are founded upon publicly shared data and remain accessible for public scrutiny in accordance with the Wiley Research Data Policy.
