# Supplementary material for: Thriving at Work Among Clinical Nurses: A Systematic Review and Meta‐Analysis
Source: J Nurs Manag. 2026 Oct 28;2026:9263276. doi: 10.1155/jonm/9263276 (PMC13714573; doi:10.1155/jonm/9263276)
Supplement: Supplementary file 1 [file JONM-2026-9263276-s001.zip › Supporting Information Figure S5.pdf]

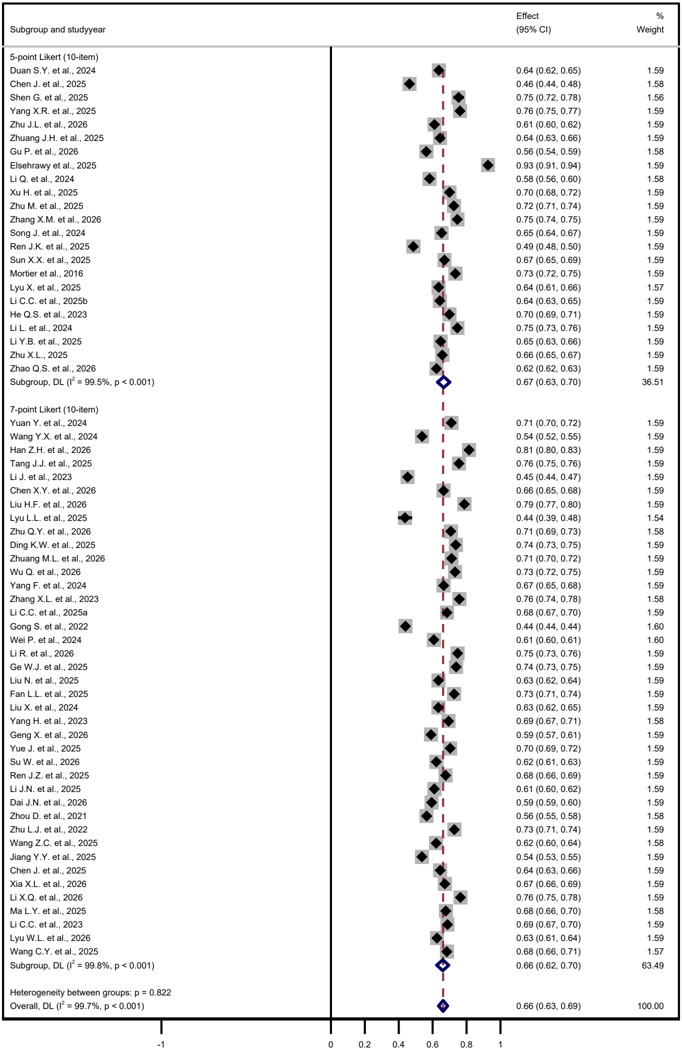

NOTE: Weights and between-subgroup heterogeneity test are from random-effects model
