# Supplementary material for: Thriving at Work Among Clinical Nurses: A Systematic Review and Meta‐Analysis
Source: J Nurs Manag. 2026 Oct 28;2026:9263276. doi: 10.1155/jonm/9263276 (PMC13714573; doi:10.1155/jonm/9263276)
Supplement: Supplementary file 1 [file JONM-2026-9263276-s001.zip › Supporting Information Figure S6.pdf]

subg_career and studyyear	Effect (95% CI)	% Weight
**Early-career nurses**		
Han Z.H. et al., 2026	0.81 (0.80, 0.83)	1.57
Chen J. et al., 2025	0.46 (0.44, 0.48)	1.56
Yuan Y. et al., 2024	0.71 (0.70, 0.72)	1.57
Wang Y.X. et al., 2024	0.54 (0.52, 0.55)	1.56
Duan S.Y. et al., 2024	0.64 (0.62, 0.65)	1.56
Zhu J.L. et al., 2026	0.61 (0.60, 0.62)	1.57
Yang X.R. et al., 2025	0.76 (0.75, 0.77)	1.57
Shen G. et al., 2025	0.75 (0.72, 0.78)	1.54
Subgroup, DL (I^2 = 99.6%, $p < 0.001$)	0.66 (0.59, 0.73)	12.49
**Mixed-seniority nurses**		
Zhuang M.L. et al., 2026	0.71 (0.70, 0.72)	1.57
Liu X. et al., 2024	0.63 (0.62, 0.65)	1.56
Yang F. et al., 2024	0.67 (0.65, 0.68)	1.56
Geng X. et al., 2026	0.59 (0.57, 0.61)	1.56
Tang J.J. et al., 2025	0.76 (0.75, 0.76)	1.57
Atalla et al., 2024	0.65 (0.65, 0.66)	1.57
Ge W.J. et al., 2025	0.74 (0.73, 0.75)	1.56
Fan L.L. et al., 2025	0.73 (0.71, 0.74)	1.56
Chen J. et al., 2025	0.64 (0.63, 0.66)	1.57
Li C.C. et al., 2025a	0.68 (0.67, 0.70)	1.56
Zhu L.J. et al., 2022	0.73 (0.71, 0.74)	1.56
Ren J.K. et al., 2025	0.49 (0.48, 0.50)	1.57
Lyu L.L. et al., 2025	0.44 (0.39, 0.48)	1.51
He Q.S. et al., 2023	0.70 (0.69, 0.71)	1.57
Chen X.Y. et al., 2026	0.66 (0.65, 0.68)	1.57
Yue J. et al., 2025	0.70 (0.69, 0.72)	1.57
Zhu M. et al., 2025	0.72 (0.71, 0.74)	1.56
Zhao Q.S. et al., 2026	0.62 (0.62, 0.63)	1.57
Li Y.B. et al., 2025	0.65 (0.63, 0.66)	1.57
Wei P. et al., 2024	0.61 (0.60, 0.61)	1.57
Yang H. et al., 2023	0.69 (0.67, 0.71)	1.56
Zhou D. et al., 2021	0.56 (0.55, 0.58)	1.56
Liu N. et al., 2025	0.63 (0.62, 0.64)	1.57
Li X.Q. et al., 2026	0.76 (0.75, 0.78)	1.56
Xu H. et al., 2025	0.70 (0.68, 0.72)	1.56
Ding K.W. et al., 2025	0.74 (0.73, 0.75)	1.57
Lyu W.L. et al., 2026	0.63 (0.61, 0.64)	1.56
Wu Q. et al., 2026	0.73 (0.72, 0.75)	1.56
Li J.N. et al., 2025	0.61 (0.60, 0.62)	1.57
Zhu Q.Y. et al., 2026	0.71 (0.69, 0.73)	1.56
Zhang X.M. et al., 2026	0.75 (0.74, 0.75)	1.57
Wang Z.C. et al., 2025	0.62 (0.60, 0.64)	1.55
Zhuang J.H. et al., 2025	0.64 (0.63, 0.66)	1.56
Gong S. et al., 2022	0.44 (0.44, 0.44)	1.57
Li Q. et al., 2024	0.58 (0.56, 0.60)	1.56
Li L. et al., 2024	0.75 (0.73, 0.76)	1.57
Sun X.X. et al., 2025	0.67 (0.65, 0.69)	1.56
Li R. et al., 2026	0.75 (0.73, 0.76)	1.56
Lyu X. et al., 2025	0.64 (0.61, 0.66)	1.55
Ren J.Z. et al., 2025	0.68 (0.66, 0.69)	1.56
Li C.C. et al., 2023	0.69 (0.67, 0.70)	1.56
Xia X.L. et al., 2026	0.67 (0.66, 0.69)	1.56
Li J. et al., 2023	0.45 (0.44, 0.47)	1.56
Song J. et al., 2024	0.65 (0.64, 0.67)	1.56
Gu P. et al., 2026	0.56 (0.54, 0.59)	1.55
Su W. et al., 2026	0.62 (0.61, 0.63)	1.57
Wang C.Y. et al., 2025	0.68 (0.66, 0.71)	1.55
Zhang X.L. et al., 2023	0.76 (0.74, 0.78)	1.56
Li C.C. et al., 2025b	0.64 (0.63, 0.65)	1.57
Dai J.N. et al., 2026	0.59 (0.59, 0.60)	1.57
Liu H.F. et al., 2026	0.79 (0.77, 0.80)	1.56
Ma L.Y. et al., 2025	0.68 (0.66, 0.70)	1.55
Jiang Y.Y. et al., 2025	0.54 (0.53, 0.55)	1.57
Elsehrawy et al., 2025	0.93 (0.91, 0.94)	1.56
Mortier et al., 2016	0.73 (0.72, 0.75)	1.57
Zhu X.L., 2025	0.66 (0.65, 0.67)	1.57
Subgroup, DL (I^2 = 99.7%, $p < 0.001$)	0.66 (0.63, 0.69)	87.51
Heterogeneity between groups: $p = 0.986$		
Overall, DL (I^2 = 99.7%, $p < 0.001$)	0.66 (0.64, 0.69)	100.00

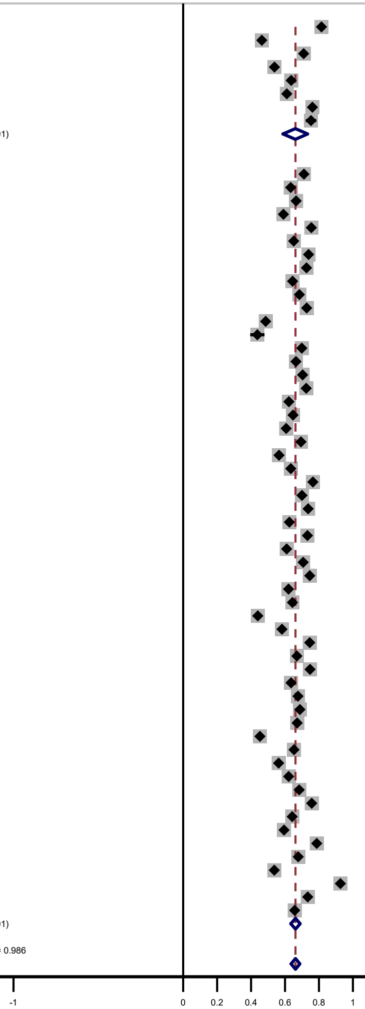

NOTE: Weights and between-subgroup heterogeneity test are from random-effects model
