# Supplementary material for: Thriving at Work Among Clinical Nurses: A Systematic Review and Meta‐Analysis
Source: J Nurs Manag. 2026 Oct 28;2026:9263276. doi: 10.1155/jonm/9263276 (PMC13714573; doi:10.1155/jonm/9263276)
Supplement: Supplementary file 1 [file JONM-2026-9263276-s001.zip › Supporting Information Figure S7.pdf]

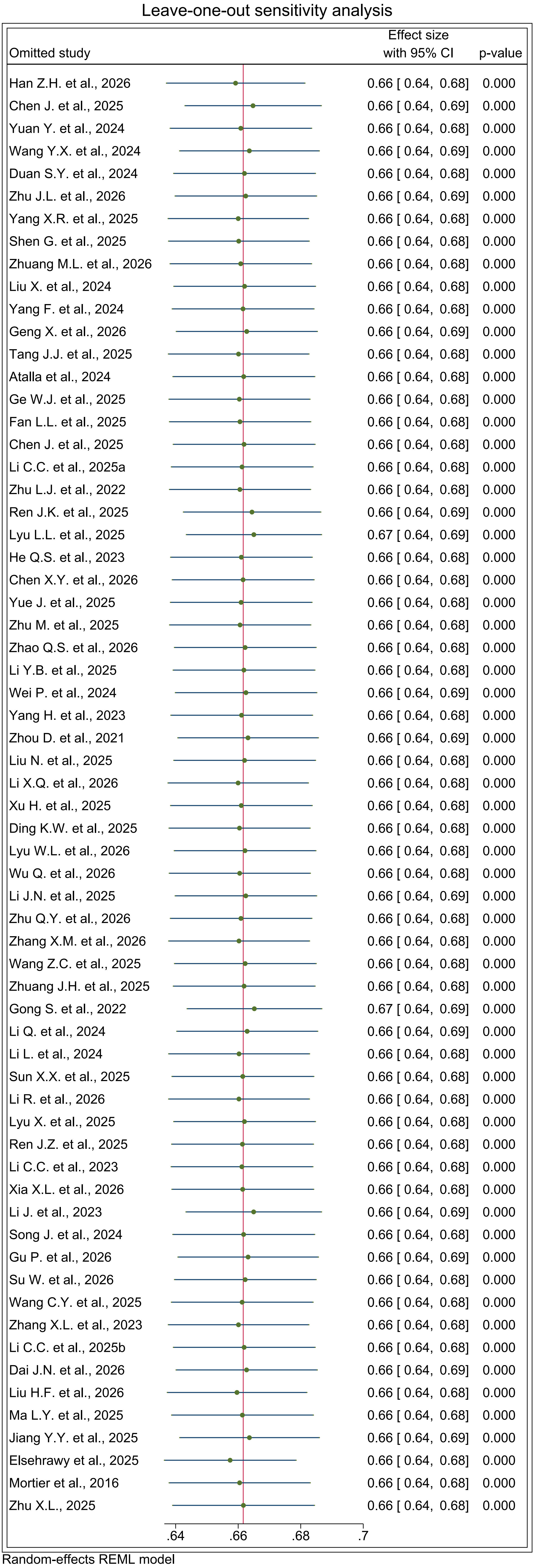
Leave-one-out sensitivity analysis
Omitted study
Effect size with 95% CI
p-value
Han Z.H. et al., 2026 0.66 [0.64, 0.68] 0.000
Chen J. et al., 2025 0.66 [0.64, 0.69] 0.000
Yuan Y. et al., 2024 0.66 [0.64, 0.68] 0.000
Wang Y.X. et al., 2024 0.66 [0.64, 0.69] 0.000
Duan S.Y. et al., 2024 0.66 [0.64, 0.68] 0.000
Zhu J.L. et al., 2026 0.66 [0.64, 0.69] 0.000
Yang X.R. et al., 2025 0.66 [0.64, 0.68] 0.000
Shen G. et al., 2025 0.66 [0.64, 0.68] 0.000
Zhuang M.L. et al., 2026 0.66 [0.64, 0.68] 0.000
Liu X. et al., 2024 0.66 [0.64, 0.68] 0.000
Yang F. et al., 2024 0.66 [0.64, 0.68] 0.000
Geng X. et al., 2026 0.66 [0.64, 0.69] 0.000
Tang J.J. et al., 2025 0.66 [0.64, 0.68] 0.000
Atalla et al., 2024 0.66 [0.64, 0.68] 0.000
Ge W.J. et al., 2025 0.66 [0.64, 0.68] 0.000
Fan L.L. et al., 2025 0.66 [0.64, 0.68] 0.000
Chen J. et al., 2025 0.66 [0.64, 0.68] 0.000
Li C.C. et al., 2025a 0.66 [0.64, 0.68] 0.000
Zhu L.J. et al., 2022 0.66 [0.64, 0.68] 0.000
Ren J.K. et al., 2025 0.66 [0.64, 0.69] 0.000
Lyu L.L. et al., 2025 0.67 [0.64, 0.69] 0.000
He Q.S. et al., 2023 0.66 [0.64, 0.68] 0.000
Chen X.Y. et al., 2026 0.66 [0.64, 0.68] 0.000
Yue J. et al., 2025 0.66 [0.64, 0.68] 0.000
Zhu M. et al., 2025 0.66 [0.64, 0.68] 0.000
Zhao Q.S. et al., 2026 0.66 [0.64, 0.68] 0.000
Li Y.B. et al., 2025 0.66 [0.64, 0.68] 0.000
Wei P. et al., 2024 0.66 [0.64, 0.69] 0.000
Yang H. et al., 2023 0.66 [0.64, 0.68] 0.000
Zhou D. et al., 2021 0.66 [0.64, 0.69] 0.000
Liu N. et al., 2025 0.66 [0.64, 0.68] 0.000
Li X.Q. et al., 2026 0.66 [0.64, 0.68] 0.000
Xu H. et al., 2025 0.66 [0.64, 0.68] 0.000
Ding K.W. et al., 2025 0.66 [0.64, 0.68] 0.000
Lyu W.L. et al., 2026 0.66 [0.64, 0.68] 0.000
Wu Q. et al., 2026 0.66 [0.64, 0.68] 0.000
Li J.N. et al., 2025 0.66 [0.64, 0.69] 0.000
Zhu Q.Y. et al., 2026 0.66 [0.64, 0.68] 0.000
Zhang X.M. et al., 2026 0.66 [0.64, 0.68] 0.000
Wang Z.C. et al., 2025 0.66 [0.64, 0.68] 0.000
Zhuang J.H. et al., 2025 0.66 [0.64, 0.68] 0.000
Gong S. et al., 2022 0.67 [0.64, 0.69] 0.000
Li Q. et al., 2024 0.66 [0.64, 0.69] 0.000
Li L. et al., 2024 0.66 [0.64, 0.68] 0.000
Sun X.X. et al., 2025 0.66 [0.64, 0.68] 0.000
Li R. et al., 2026 0.66 [0.64, 0.68] 0.000
Lyu X. et al., 2025 0.66 [0.64, 0.68] 0.000
Ren J.Z. et al., 2025 0.66 [0.64, 0.68] 0.000
Li C.C. et al., 2023 0.66 [0.64, 0.68] 0.000
Xia X.L. et al., 2026 0.66 [0.64, 0.68] 0.000
Li J. et al., 2023 0.66 [0.64, 0.69] 0.000
Song J. et al., 2024 0.66 [0.64, 0.68] 0.000
Gu P. et al., 2026 0.66 [0.64, 0.69] 0.000
Su W. et al., 2026 0.66 [0.64, 0.68] 0.000
Wang C.Y. et al., 2025 0.66 [0.64, 0.68] 0.000
Zhang X.L. et al., 2023 0.66 [0.64, 0.68] 0.000
Li C.C. et al., 2025b 0.66 [0.64, 0.68] 0.000
Dai J.N. et al., 2026 0.66 [0.64, 0.69] 0.000
Liu H.F. et al., 2026 0.66 [0.64, 0.68] 0.000
Ma L.Y. et al., 2025 0.66 [0.64, 0.68] 0.000
Jiang Y.Y. et al., 2025 0.66 [0.64, 0.69] 0.000
Elsehrawy et al., 2025 0.66 [0.64, 0.68] 0.000
Mortier et al., 2016 0.66 [0.64, 0.68] 0.000
Zhu X.L., 2025 0.66 [0.64, 0.68] 0.000
.64
.66
.68
.7
Random-effects REML model
