# Supplementary material for: Thriving at Work Among Clinical Nurses: A Systematic Review and Meta‐Analysis
Source: J Nurs Manag. 2026 Oct 28;2026:9263276. doi: 10.1155/jonm/9263276 (PMC13714573; doi:10.1155/jonm/9263276)
Supplement: Supplementary file 1 [file JONM-2026-9263276-s001.zip › Supporting Information Figure S8.pdf]

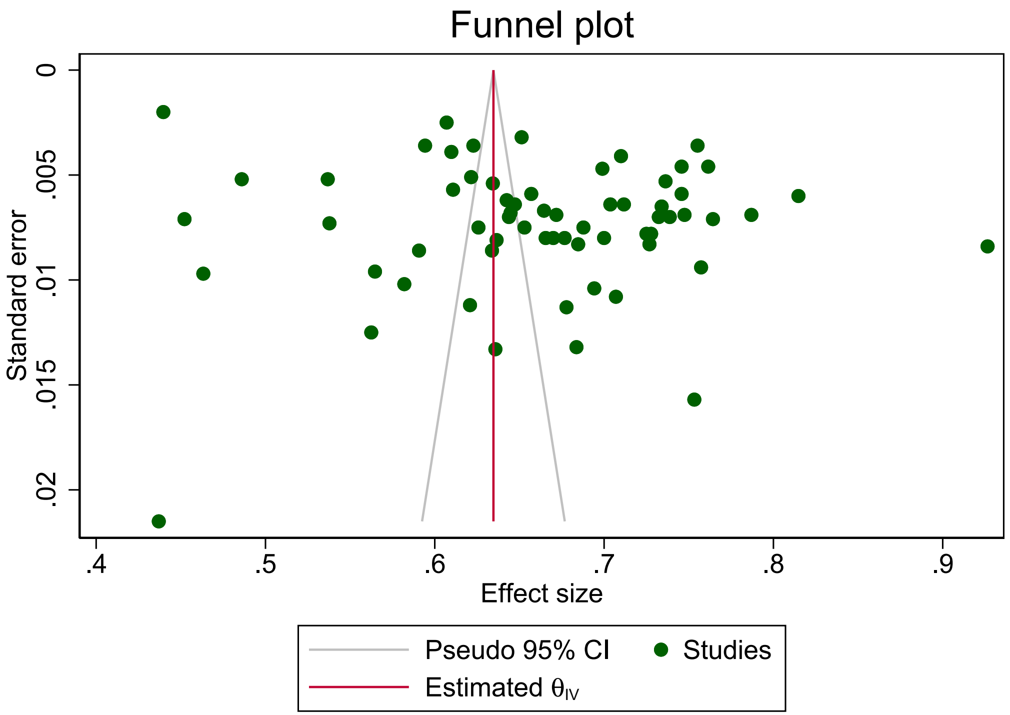

Funnel plot
Standard error
0
.005
.01
.015
.02
.4
.5
.6
.7
.8
.9
Effect size
Pseudo 95% CI
Studies
Estimated θ_{IV}
