# Supplementary material for: Thriving at Work Among Clinical Nurses: A Systematic Review and Meta‐Analysis
Source: J Nurs Manag. 2026 Oct 28;2026:9263276. doi: 10.1155/jonm/9263276 (PMC13714573; doi:10.1155/jonm/9263276)
Supplement: Supplementary file 1 [file JONM-2026-9263276-s001.zip › Supporting Information.docx]

**Supporting Information**

**Table S1. Search strategies for all databases**

| **Database** | **Search terms** | **Results** |
| --- | --- | --- |
| PubMed | #1 "Nurses"[Mesh] OR "nurse"[Title/Abstract] OR "nursing personnel"[Title/Abstract] OR "personnel, nursing"[Title/Abstract] OR "registered nurses"[Title/Abstract] OR "nurse, registered"[Title/Abstract] OR "nurses, registered"[Title/Abstract] OR "registered nurse"[Title/Abstract] OR "nurs*"[Title/Abstract]  #2 "Work"[MeSH Terms] OR "work"[Title/Abstract] OR "works"[Title/Abstract] OR "working"[Title/Abstract] OR "worked"[Title/Abstract] OR "job"[Title/Abstract] OR "jobs"[Title/Abstract] OR "task"[Title/Abstract] OR "tasks"[Title/Abstract]  #3 "thrive"[Title/Abstract] OR "thrives"[Title/Abstract] OR "thriving"[Title/Abstract] OR "thrived"[Title/Abstract] OR "flourish"[Title/Abstract] OR "flourishing"[Title/Abstract] OR "flourished"[Title/Abstract] OR "flourishes"[Title/Abstract]  #4 #2 AND #3  #5 "thriving at work"[Title/Abstract]  #6 #4 OR #5  #7 #1 AND #6 | 335 |
| Cochrane Library | #1 (nurse):ab,ti,kw OR (nursing personnel):ab,ti,kw OR (personnel nursing):ab,ti,kw OR (registered nurses):ab,ti,kw OR (nurse registered):ab,ti,kw OR (nurses registered):ab,ti,kw OR (registered nurse):ab,ti,kw OR (nurs*):ab,ti,kw  #2 (work):ab,ti,kw OR (works):ab,ti,kw OR (working):ab,ti,kw OR (worked):ab,ti,kw OR (job):ab,ti,kw OR (jobs):ab,ti,kw OR (task):ab,ti,kw OR (tasks):ab,ti,kw  #3 (thrive):ab,ti,kw OR (thrives):ab,ti,kw OR (thriving):ab,ti,kw OR (thrived):ab,ti,kw OR (flourish):ab,ti,kw OR (flourishing):ab,ti,kw OR (flourished):ab,ti,kw OR (flourishes):ab,ti,kw  #4 #2 AND #3  #5 ("thriving at work"):ab,ti,kw  #6 #4 OR #5  #7 #1 AND #6 | 53 |
| Embase | #1 'nurses':ab,ti OR 'nursing personnel':ab,ti OR 'personnel nursing':ab,ti OR 'registered nurses':ab,ti OR 'nurse registered':ab,ti OR 'nurses registered':ab,ti OR 'registered nurse':ab,ti OR 'nurs*':ab,ti  #2 'work':ab,ti OR 'works':ab,ti OR 'working':ab,ti OR 'worked':ab,ti OR 'job':ab,ti OR 'jobs':ab,ti OR 'task':ab,ti OR 'tasks':ab,ti  #3 'thrive':ab,ti OR 'thrives':ab,ti OR 'thriving':ab,ti OR 'thrived':ab,ti OR 'flourish':ab,ti OR 'flourishing':ab,ti OR 'flourished':ab,ti OR 'flourishes':ab,ti  #4 #2 AND #3  #5 'thriving at work':ab,ti  #6 #4 OR #5  #7 #1 AND #6 | 678 |
| Web of Science | #1 TS=("nurses" OR "nurse" OR "nursing personnel" OR "personnel nursing" OR "registered nurses" OR "nurse registered" OR "nurses registered" OR "registered nurse" OR "nurs*")  #2 TS=("work" OR "works" OR "working" OR "worked" OR "job" OR "jobs" OR "task" OR "tasks")  #3 TS=("thrive" OR "thrives" OR "thriving" OR "thrived" OR "flourish" OR "flourishing" OR "flourished" OR "flourishes")  #4 #2 AND #3  #5 TS=("thriving at work")  #6 #4 OR #5  #7 #1 AND #6 | 349 |
| CNKI | #1 SU=(护士OR临床护士OR护理人员OR注册护士)  #2 SU=(工作繁荣OR工作旺盛感OR工作成长)  #3 #1 AND #2 | 97 |
| Wanfang | #1 (主题:("护士") OR 主题:("临床护士") OR 主题:("护理人员") OR 主题:("注册护士"))  #2 (主题:("工作繁荣") OR 主题:("工作旺盛感") OR 主题:("工作成长"))  #3 #1 AND #2 | 397 |
| VIP | #1 M=(护士 OR 临床护士 OR 护理人员 OR 注册护士)  #2 M=(工作繁荣 OR 工作旺盛感 OR 工作成长)  #3 #1 AND #2 | 77 |
| CBM | #1 ("护士"[常用字段:智能] OR "临床护士"[常用字段:智能] OR "注册护士"[常用字段:智能] OR "护理人员"[常用字段:智能])  #2 ("工作繁荣"[常用字段:智能] OR "工作旺盛感"[常用字段:智能] OR "工作成长"[常用字段:智能])  #3 #1 AND #2 | 51 |

**Table S2. Quality assessment results of included studies.**

| **Author, year** | **Item 1** | **Item 2** | **Item 3** | **Item 4** | **Item 5** | **Item 6** | **Item 7** | **Item 8** | **Item 9** | **Item 10** | **Item 11** | **Total score** | **Quality level** |
| --- | --- | --- | --- | --- | --- | --- | --- | --- | --- | --- | --- | --- | --- |
| He Q.S. et al., 2023 | Y | Y | Y | U | U | Y | Y | Y | Y | Y | U | 8 | High |
| Wang Z.C. et al., 2025 | Y | Y | Y | U | U | Y | N | Y | U | Y | U | 6 | Moderate |
| Li C.C. et al., 2023 | Y | Y | Y | U | U | Y | Y | Y | U | Y | U | 7 | Moderate |
| Yang H. et al., 2023 | Y | Y | Y | U | U | Y | Y | Y | U | Y | U | 7 | Moderate |
| Wei P. et al., 2024 | Y | Y | Y | U | U | Y | Y | Y | Y | Y | U | 8 | High |
| Song J. et al., 2024 | Y | Y | Y | U | U | Y | Y | Y | U | Y | U | 7 | Moderate |
| Lyu X. et al., 2025a | Y | Y | Y | U | U | Y | Y | Y | U | Y | U | 7 | Moderate |
| Ren J.Z. et al., 2025 | Y | Y | Y | U | U | Y | Y | Y | U | Y | U | 7 | Moderate |
| Zhu L.J. et al., 2022 | Y | Y | Y | U | U | Y | N | Y | U | Y | U | 6 | Moderate |
| Zhou D. et al., 2021 | Y | Y | Y | U | U | Y | N | Y | U | Y | U | 6 | Moderate |
| Fan L.L. et al., 2025 | Y | Y | Y | Y | U | Y | N | Y | U | Y | U | 7 | Moderate |
| Jiang Y.Y. et al., 2025 | Y | Y | Y | U | U | Y | Y | Y | U | Y | U | 7 | Moderate |
| Yang F. et al., 2024 | Y | Y | Y | Y | U | Y | Y | Y | U | Y | U | 8 | High |
| Zhu M. et al., 2025 | Y | Y | Y | U | U | Y | N | Y | U | Y | U | 6 | Moderate |
| Zhang X.L. et al., 2023 | Y | Y | Y | U | U | Y | Y | Y | Y | Y | U | 8 | High |
| Li L. et al., 2024 | Y | Y | Y | U | U | Y | N | Y | U | Y | U | 6 | Moderate |
| Li Q. et al., 2024 | Y | Y | Y | U | U | Y | Y | Y | U | Y | U | 7 | Moderate |
| Ma L.Y. et al., 2025 | Y | Y | Y | U | U | Y | Y | Y | U | Y | U | 7 | Moderate |
| Ding K.W. et al., 2025 | Y | Y | Y | U | U | Y | Y | N | Y | Y | U | 7 | Moderate |
| Li C.C. et al., 2025a | Y | Y | Y | U | U | Y | N | Y | U | Y | U | 6 | Moderate |
| Liu X. et al., 2024 | Y | Y | Y | U | U | Y | N | Y | U | Y | U | 6 | Moderate |
| Li J. et al., 2023 | Y | Y | Y | U | U | Y | N | Y | U | Y | U | 6 | Moderate |
| Gong S. et al., 2022 | Y | Y | Y | Y | U | Y | N | Y | U | Y | U | 7 | Moderate |
| Sun X.X. et al., 2025 | Y | Y | Y | U | U | Y | Y | Y | U | Y | U | 7 | Moderate |
| Lyu L.L. et al., 2025 | Y | Y | Y | U | U | Y | Y | Y | Y | Y | U | 8 | High |
| Duan S.Y. et al., 2024 | Y | Y | Y | U | U | Y | Y | Y | Y | Y | U | 8 | High |
| Wang Y.X. et al., 2024 | Y | Y | Y | U | U | Y | Y | N | Y | Y | U | 7 | Moderate |
| Yang X.R. et al., 2025 | Y | Y | Y | U | U | Y | Y | Y | U | Y | U | 7 | Moderate |
| Yuan Y. et al., 2024 | Y | Y | Y | U | U | Y | Y | Y | Y | Y | U | 8 | High |
| Mortier et al., 2016 | Y | N | Y | U | U | Y | N | Y | U | Y | U | 5 | Moderate |
| Atalla et al., 2024 | Y | Y | N | Y | U | Y | N | Y | U | N | U | 5 | Moderate |
| Shen G. et al., 2025 | Y | Y | Y | U | U | Y | Y | Y | Y | Y | U | 8 | High |
| Zhu J.L. et al., 2026 | Y | Y | Y | U | U | Y | N | Y | U | N | U | 5 | Moderate |
| Xia X.L. et al., 2026 | Y | Y | Y | U | U | Y | Y | Y | U | Y | U | 7 | Moderate |
| Tang J.J. et al., 2025 | Y | Y | Y | U | U | Y | Y | Y | Y | Y | U | 8 | High |
| Wang C.Y. et al., 2025 | Y | Y | Y | U | U | Y | N | Y | U | Y | U | 6 | Moderate |
| Geng X. et al., 2026 | Y | Y | Y | U | U | Y | Y | Y | Y | Y | U | 8 | High |
| Ren J.K. et al., 2025 | Y | Y | Y | U | U | Y | Y | Y | U | Y | U | 7 | Moderate |
| Liu H.F. et al., 2026 | Y | Y | Y | U | U | Y | Y | Y | U | Y | U | 7 | Moderate |
| Gu P. et al., 2026 | Y | Y | Y | U | U | Y | N | Y | U | Y | U | 6 | Moderate |
| Yue J. et al., 2025 | Y | Y | Y | U | U | Y | N | Y | U | Y | U | 6 | Moderate |
| Li R. et al., 2026 | Y | Y | Y | Y | U | Y | Y | Y | U | Y | U | 8 | High |
| Li X.Q. et al., 2026 | Y | Y | Y | U | U | Y | Y | Y | U | Y | U | 7 | Moderate |
| Zhu X.L., 2025 | Y | Y | Y | U | U | Y | Y | Y | U | Y | U | 7 | Moderate |
| Li Y.B. et al., 2025 | Y | Y | Y | U | U | Y | Y | Y | Y | Y | U | 8 | High |
| Chen J. et al., 2025 | Y | Y | Y | U | U | Y | N | Y | U | N | U | 5 | Moderate |
| Su W. et al., 2026 | Y | Y | Y | U | U | Y | N | Y | U | Y | U | 6 | Moderate |
| Zhuang M.L. et al., 2026 | Y | Y | Y | U | U | Y | Y | Y | U | Y | U | 7 | Moderate |
| Chen J. et al., 2025 | Y | Y | Y | U | U | Y | N | Y | U | Y | U | 6 | Moderate |
| Li C.C. et al., 2025 | Y | Y | Y | U | U | Y | Y | Y | Y | Y | U | 8 | High |
| Dai J.N. et al., 2026 | Y | Y | Y | U | U | Y | Y | Y | U | Y | U | 7 | Moderate |
| Ge W.J. et al., 2025 | Y | Y | N | U | U | Y | Y | Y | Y | Y | U | 7 | Moderate |
| Chen X.Y. et al., 2026 | Y | Y | Y | U | U | Y | Y | Y | U | Y | U | 7 | Moderate |
| Lyu W.L. et al., 2026 | Y | Y | Y | U | U | Y | Y | Y | U | Y | U | 7 | Moderate |
| Han Z.H. et al., 2026 | Y | Y | Y | U | U | Y | Y | Y | Y | Y | U | 8 | High |
| Zhang X.M. et al., 2026 | Y | Y | Y | U | U | Y | Y | Y | U | Y | U | 7 | Moderate |
| Zhu Q.Y. et al., 2026 | Y | Y | Y | Y | U | Y | Y | Y | Y | Y | U | 9 | High |
| Liu N. et al., 2025 | Y | Y | Y | U | U | Y | N | Y | U | Y | U | 6 | Moderate |
| Wu Q. et al., 2026 | Y | Y | Y | U | U | Y | Y | Y | Y | Y | U | 8 | High |
| Zhuang J.H. et al., 2025 | Y | Y | N | U | U | Y | Y | Y | U | Y | U | 6 | Moderate |
| Li J.N. et al., 2025 | Y | Y | Y | U | U | Y | Y | Y | Y | N | U | 7 | Moderate |
| Xu H. et al., 2025 | Y | Y | Y | U | U | Y | Y | Y | Y | N | U | 7 | Moderate |
| Zhao Q.S. et al., 2026 | Y | Y | Y | U | U | Y | Y | Y | Y | Y | U | 8 | High |
| Elsehrawy et al., 2025 | Y | Y | Y | U | U | Y | Y | Y | Y | Y | U | 8 | High |

Note: Y, yes; N, no; U, unclear. Item1, Define the source of information; Item 2, List inclusion and exclusion criteria; Item 3, Indicate time period used for identifying patients; Item 4, Indicate whether or not subjects were consecutive; Item 5, Indicate if evaluators were masked; Item 6, Describe quality assurance assessments; Item 7, Explain patient exclusions from analysis; Item 8, Describe how confounding was assessed/controlled; Item 9, Explain missing data handling; Item 10, Summarize patient response rates and completeness; Item 11, Clarify follow-up and proportion of missing data.

**Table S3. Univariate meta-regression analysis results**

| Covariate | Comparison | β (95% CI) | SE | z | P | Overall P | R² (%) | No. of studies |
| --- | --- | --- | --- | --- | --- | --- | --- | --- |
| Sample size | ≥400 vs <400 | -0.0007 (-0.0463, 0.0448) | 0.0232 | -0.03 | 0.974 | 0.974 | 0.00 | 64 |
| Study region | Eastern vs Central | 0.0392 (-0.0119, 0.0902) | 0.0260 | 1.50 | 0.132 | 0.017 | 10.81 | 62 |
|  | Overseas vs Central | 0.1462 (0.0420, 0.2504) | 0.0532 | 2.75 | 0.006 |  |  |  |
|  | Western vs Central | 0.0644 (0.0073, 0.1216) | 0.0292 | 2.21 | 0.027 |  |  |  |
| Publication year | Continuous | 0.0049 (-0.0095, 0.0193) | 0.0074 | 0.67 | 0.502 | 0.502 | 0.00 | 64 |
| Clinical department | Multi vs Critical care | 0.0158 (-0.0509, 0.0826) | 0.0340 | 0.47 | 0.642 | 0.542 | 0.00 | 64 |
|  | Operating room vs Critical care | 0.0487 (-0.0461, 0.1435) | 0.0484 | 1.01 | 0.314 |  |  |  |
|  | Other specialized vs Critical care | -0.0152 (-0.0960, 0.0656) | 0.0412 | -0.37 | 0.712 |  |  |  |
| Scale version | 7-point vs 5-point Likert | -0.0058 (-0.0532, 0.0417) | 0.0242 | -0.24 | 0.812 | 0.812 | 0.00 | 63 |
| Career stage | Mixed-seniority vs Early-career | 0.0007 (-0.0673, 0.0687) | 0.0347 | 0.02 | 0.983 | 0.983 | 0.00 | 64 |

**Figure S1. Forest plot of subgroup analysis by sample size.**

**Figure S2. Forest plot of subgroup analysis by study region.**

**Figure S3. Forest plot of subgroup analysis by publication year.**

**Figure S4. Forest plot of subgroup analysis by department.**

**Figure S5. Forest plot of subgroup analysis by scale version.**

**Figure S6. Forest plot of subgroup analysis by career stage.**

**Figure S7. Leave-one-out sensitivity analysis.**

**Figure S8. Funnel plot for publication bias assessment.**

**Figure S9. Trim-and-fill adjusted funnel plot.**
